# Primary aldosteronism-induced hypokalemic rhabdomyolysis syndrome: a case report and literature review

**DOI:** 10.3389/fmed.2026.1783683

**Published:** 2026-06-23

**Authors:** Luping Cheng, Chuanliang Pan, Koushu Wang, Wenxin Wang, Xia Hu, Hong Wan, Bo Zhang, Ying Shu

**Affiliations:** 1 Department of Nephrology, The Third People's Hospital of Chengdu, Affiliated Hospital of Southwest Jiaotong University, Chengdu, China; 2 Department of Intensive Care Unit, The Third People's Hospital of Chengdu, Affiliated Hospital of Southwest Jiaotong University, Chengdu, China; 3 North Sichuan Medical College, Nanchong, Sichuan, China

**Keywords:** adrenal adenoma, case report, hypokalemia, primary aldosteronism, rhabdomyolysis

## Abstract

**Background:**

Cases of Primary aldosteronism (PA) complicated by rhabdomyolysis (RM) are extremely rare. If not promptly recognized and managed, this condition may trigger severe muscle damage, leading to acute kidney injury (AKI), disseminated intravascular coagulation (DIC), and other serious complications, potentially resulting in death.

**Case presentation:**

We report a rare case of rhabdomyolysis characterized by recurrent generalized pain and weakness after mild exertion. Laboratory tests consistently revealed markedly elevated creatine kinase levels with hypokalemia. The underlying cause was primary aldosteronism, in which spontaneous, refractory hypokalemia led to rhabdomyolysis.

**Conclusions:**

This case highlights the importance of early recognition of PA-associated hypokalemia to prevent RM and its complications. Timely diagnosis and treatment are essential to improve outcomes and guide clinicians in managing similar cases.

## Background

Rhabdomyolysis (RM) is a clinical syndrome caused by damage to the integrity of skeletal muscle cell membranes, leading to the release of substances such as creatine kinase (CK) and myoglobin (Mb) into the bloodstream and triggering a series of metabolic disorders. Though its incidence is low, it often presents with severe clinical manifestations, and some patients may develop life-threatening complications like acute kidney injury (AKI), disseminated intravascular coagulation (DIC), and arrhythmias. The etiology is diverse, including trauma, high-intensity exercise, drug toxicity, electrolyte disturbances, endocrine and autoimmune diseases, and genetic metabolic disorders (1–3). Hypokalemia, though rare, is a significant precipitating factor that is often overlooked in clinical practice. It can disrupt muscle cell membrane potential and energy metabolism, thereby increasing muscle cells' susceptibility to injury and triggering the onset of RM. Hypokalemia has complex etiologies, commonly including gastrointestinal potassium loss, diuretic use, and renal tubular abnormalities. Among these, primary aldosteronism (PA) is a key endocrine cause of hypokalemia, arising from excessive aldosterone secretion that increases potassium excretion in the distal tubules (4). Hypokalemia related to PA often has characteristics of spontaneity, persistence, and refractory, and it is very rare for PA-induced hypokalemia to be complicated by RM. If not promptly recognized and managed, it may trigger severe muscle damage, leading to AKI, DIC, and other life-threatening complications, potentially resulting in death. This article reports a case of RM caused by spontaneous refractory hypokalemia, with the hypokalemia resulting from PA. The purpose of this article is to raise clinical physicians' awareness of RM related to this etiology, emphasize the importance of early identification and intervention in preventing RM, and familiarize them with the diagnosis and treatment process of PA secondary RM.

## Case presentation

### General information

Patient, female, 41 years old, presented to our hospital in early 2025 with “generalized pain and weakness for 4 months, recurrence for 5 days.” The patient reported that the symptoms first appeared 5 months ago, initially experiencing muscle and lower back soreness after an outing. The activity involved hiking but mainly using a cable car, with very little actual walking and no significant physical exertion. She also experienced general weakness but no chills, fever, dizziness, headache, cough with sputum, chest tightness or pain, nausea, vomiting, hematuria, or tea-colored urine. She had previously visited the emergency department (ED) of a tertiary hospital, where symptoms improved and did not recur, and she did not follow up. Five days ago, the patient experienced recurrent lower back pain after lifting everyday items (not heavy), with symptoms gradually spreading to the limbs, particularly the knee joint areas, accompanied by general weakness. The patient sought treatment at the local hospital, but symptoms showed no significant improvement. For further diagnosis and treatment, the patient was subsequently admitted to our hospital. The patient has a history of hypertension and has been intermittently taking irbesartan hydrochlorothiazide tablets for blood pressure (BP) control, with unknown BP control status. The patient denies a history of diabetes, coronary heart disease, rheumatic immune diseases, thyroid disease, or liver disease. There is no history of heavy alcohol consumption, trauma, or exposure to toxins or medications. Menstrual history is normal. The patient is married with children, who are in good health. There is no family history of genetic diseases. Physical examination: temperature 36.2 °C, pulse 64 beats per minute, respiration 18 breaths per minute, BP 130/86 mmHg. The patient is alert, with a soft neck and no rigidity. Muscle strength and tone are normal in all limbs. There is no edema in the lower limbs. Physiological reflexes are present, and pathological reflexes are not elicited.

### Relevant past medical visits

Approximately 5 months prior to admission, the patient presented to the ED of a tertiary hospital with complaints of “generalized pain and weakness.” Laboratory results showed Aspartate aminotransferase (AST) 571.65 U/L, Alanine aminotransferase (ALT) 93.20 U/L, serum potassium 2.08 mmol/L, creatine kinase (CK) 27,255.0 U/L, creatine kinase isoenzyme MB (CK-MB) 703.0 U/L, myoglobin (Mb) 1,145 ng/ml. Arterial blood gas (ABG) analysis showed metabolic alkalosis (pH of 7.57, partial pressure of carbon dioxide (P_a_CO_2_) 38.0 mmHg, bicarbonate (${\mathrm{HCO}}_{3}^{\text{-}}$) 34.8 mmol/L, and base excess (BE) 11.8 mmol/L). Coagulation function was normal. The initial diagnosis was RM; severe hypokalemia; liver function impairment; and metabolic alkalosis. After intravenous fluid, potassium supplementation [oral and intravenous (IV)], hepatoprotective, and symptomatic treatment, CK and Mb levels gradually decreased, serum potassium normalized, and symptoms resolved, allowing for discharge.

The patient experienced symptom recurrence approximately 1 week prior to admission after lifting routine household items. She was admitted to a local hospital approximately 3 days later. Laboratory tests showed CK >4,000 U/L, CK-MB 99 U/L, ALT 63 U/L, AST 199 U/L, and serum potassium 2.32 mmol/L. On the day prior to admission, ABG revealed metabolic alkalosis (pH 7.578; P_a_CO_2_ 33.60 mmHg; ${\mathrm{HCO}}_{3}^{-}$ 33.0 mmol/L; BE 9.3 mmol/L). Routine blood, renal function, erythrocyte sedimentation rate (ESR), immunological tests, and tumor markers were all normal. Abdominal and pelvic computed tomography (CT) revealed a left ovarian mass and right renal calculi. Preliminary clinical considerations included possible polymyositis and RM, along with laboratory-confirmed hypokalemia and metabolic alkalosis. Intravenous fluid, potassium supplementation, and methylprednisolone 0.1 g for 3 days were administered, but muscle enzymes continued to rise. Referral to a higher-level hospital is recommended.

## Clinical course at our hospital

She was admitted to the nephrology department of our hospital in early 2025. Initial laboratory tests revealed CK 38,081U/L, CK-MB 300.3U/L, ALT 239U/L, AST 766U/L, serum potassium 3.17mmol/L. ABG indicated metabolic alkalosis (pH 7.489; P_a_CO_2_ 34.0 mmHg; ${\mathrm{HCO}}_{3}^{-}$ 26.7 mmol/L; BE 2.4 mmol/L). The ESR was elevated. Other parameters, including troponin T, brain natriuretic peptide, renal function, glycated hemoglobin, serum immunofixation electrophoresis, thyroid and sex hormones, infectious markers, and echocardiography, were within normal limits. Despite aggressive potassium supplementation, fluid resuscitation, and hepatoprotective therapy, serum potassium levels remained low. Although RM is typically associated with hyperkalemia, she presented with persistent hypokalemia across multiple tests, suggesting the possibility of hypokalemia-induced RM—a relatively rare clinical presentation. Given her history of hypertension, PA could not be excluded; however, CT scan from another hospital revealed no adrenal morphological abnormalities. Further endocrine evaluation was performed: in the upright position, aldosterone was 290.82 pg/ml, renin 3.49 pg/ml, aldosterone-renin ratio (ARR) 83.33, and angiotensin II 76.19 pg/ml. Bilateral renal and renal vascular ultrasonography showed increased parenchymal echogenicity and salt crystals in the renal sinus. 24-h (24h) ambulatory BP monitoring showed a reverse dipper pattern, with a peak of 175/113 mmHg and an average of 151/93 mmHg. Based on the patient's clinical manifestations, laboratory findings, and consultation with the endocrinology department, the following preliminary diagnostic considerations were formulated. Suspected PA was first considered: the patient had a history of hypertension and recurrent refractory hypokalemia, which strongly raised suspicion for PA. However, the patient had intermittently taken antihypertensive medications containing hydrochlorothiazide, which may also cause or aggravate hypokalemia. In addition, other potential secondary causes of hypokalemia still needed to be excluded, and further evaluation was therefore required to establish a definitive diagnosis. RM was diagnosed given that the patient presented with generalized muscle pain during this episode, and laboratory testing revealed markedly elevated CK levels, consistent with the clinical features of RM. Additional confirmed diagnoses included hypokalemia, as repeated electrolyte monitoring during the disease course consistently demonstrated decreased serum potassium levels; grade 3 hypertension, given the patient's known history of hypertension with a recorded maximum BP of 175/113 mmHg; liver function injury, suggested by markedly elevated transaminase levels on laboratory tests; and metabolic alkalosis, indicated by multiple ABG analysis. Antihypertensive therapy was changed to a non-dihydropyridine calcium channel blocker (Diltiazem Hydrochloride), while potassium supplementation and hepatoprotective therapy were continued. Adrenal CT with contrast suggested a possible left adrenal adenoma (Figure 1). Circadian rhythm of cortisol and adrenocorticotropic hormone (ACTH) and 24 h urinary electrolytes are detailed in Table 1. Plasma catecholamines and sex hormones were normal. A 1 mg overnight dexamethasone suppression test (DST) was recommended to exclude subclinical Cushing's syndrome. If necessary, adrenal vein sampling or CXCR4-targeted nuclear imaging would be performed for localization and surgical planning. Following treatment, her CK, CK-MB, AST, and ALT levels decreased significantly, and serum potassium remained stable (Figure 2). She had no significant fatigue or muscle pain and was discharged on medication: diltiazem hydrochloride 90 mg q12h, spironolactone 20 mg bid, and potassium chloride sustained-release tablets 1 g tid. Follow-up after discharge showed stable BP and serum potassium levels, with no symptoms of discomfort.

**Figure 1:**
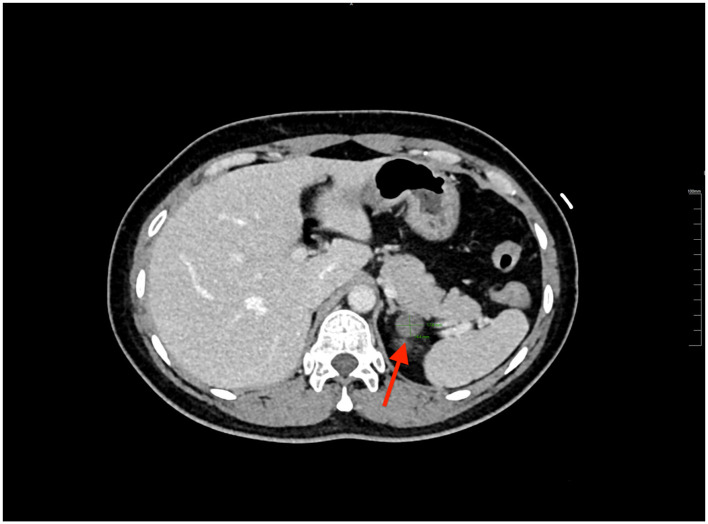
Upper abdominal (adrenal) CT scan with plain, contrast-enhanced, and 3D reconstruction revealed a low-density nodule in the left adrenal gland, measuring approximately 14.4 × 17.8 mm, demonstrating homogeneous mild enhancement on contrast imaging.

**Table 1:** Clinical variables and laboratory values while illness.

Day of illness													
Local hospital					Our hospital								
Variable	1	2	3	4	1	2	3	4	5	6	7	8	9
Clinical variables													
BP (mmHg)	–	–	–	–	130/86	128/80	125/84	131/87	135/78	140/73	145/81	142/70	130/76
24h UV (ml)	–	–	–	–	2,500	2,000	2,000	2,000	2,100	2,200	2,000	–	–
Laboratory values													
WBC ( × 10^9^/L)	9.40	–	–	–	16.82	–	10.09	–	–	–	–	–	–
RBC ( × 10^12^/L)	4.50	–	–	–	4.00	–	4.61	–	–	–	–	–	–
HGB (g/L)	104	–	–	–	104	–	122	–	–	–	–	–	–
PLT ( × 10^9^/L)	269	–	–	–	293	–	307	–	–	–	–	–	–
AST (U/L)	369	369	896	–	766	–	253	–	–	45	–	–	–
ALT (U/L)	63	–	–	–	239	–	199	–	–	91	–	–	–
LDH (U/L)	459	756	2,204	–	1,540	–	620	–	–	343	–	–	–
Cre (μmol/L)	–	50.5	49.7	–	57	–	47	–	–	64	–	–	–
K (mmol/L)	2.32	2.00	3.10	3.03	3.17	–	3.04	–	–	4.03	–	–	4.01
Na (mmol/L)	146.7	144.1	149.1	147.1	145.0	–	139.8			142.7			
Myoglobin (ng/ml)	2,128	–	–	–	1,153	–	155	–	–	31.70	–	–	–
CK (U/L)	>4,000	>4,000	>4,000	–	38,081	–	6,645	–	–	524	–	–	–
CK-MB (ng/ml)	99	160	400	–	300.3	–	80.7	–	–	19.4	–	–	–
Lac (mmol/L)	–	–	2.88	–	–	–	3.63	–	–	–	–	–	–
24h UTP (mg/24 h)	–	–	1,950	–	–	–	–	343.3	–	–	–	–	–
24h U-Na (mmol/24 h)	–	–	328.8	–	–	–	–	278.04	–	–	–	–	207.20
24h U-K (mmol/24 h)	–	–	54.0	–	–	–	–	57.65	–	–	–	–	121.14
8 am ACTH (ng/l)	–	–	–	–	–	–	–	–	–	–	–	15.30	–
4 pm ACTH (ng/l)	–	–	–	–	–	–	–	–	–	–	–	9.57	–
0 am ACTH (ng/l)	–	–	–	–	–	–	–	–	–	–	–	3.98	–
8 am cortisol (nmol/l)	–	–	–	–	–	–	–	–	–	–	–	297.00	–
4 pm cortisol (nmol/l)	–	–	–	–	–	–	–	–	–	–	–	130.00	–
0 am cortisol (nmol/l)	–	–	–	–	–	–	–	–	–	–	–	69.20	–

BP, blood pressure; WBC, white blood cell; RBC, red blood cell; HGB, hemoglobin; PLT, platelet count; AST, aspartate aminotransferase; ALT, alanine aminotransferase; LDH, lactate dehydrogenase; Cre, creatinine; K, Serum Potassium; Na, CK, creatinine kinase; CK-MB, creatine kinase isoenzyme-MB; Lac, lactate; 24h UTP, 24-hour Urine Total Protein; 24h U-Na, 24-hour Urinary Sodium; 24h U-K, 24-hour Urinary Potassium; ACTH, adrenocorticotropic hormone.

**Figure 2:**
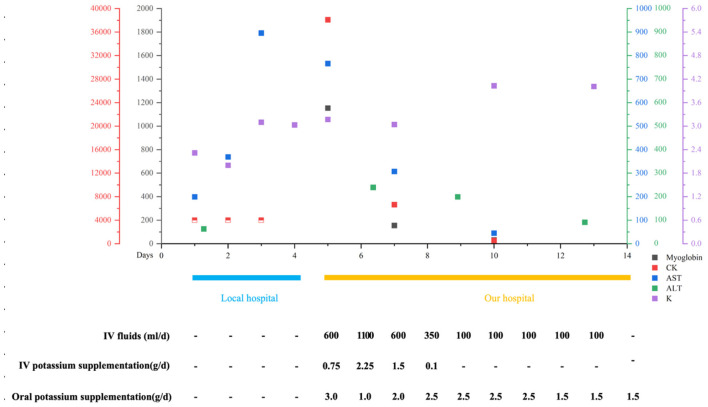
Trends of CK, Myoglobin, AST, ALT, and K Over Time. CK, creatine kinase; AST, aspartate aminotransferase; ALT, alanine transaminase; K, Serum Potassium. The three red markers 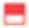 in Figure 2 indicate that the level of CK is >4,000 U/L, and the specific values are not shown.

## Clinical follow-up

Approximately 4 months later, the patient was admitted to another comprehensive tertiary hospital. On admission, comprehensive laboratory tests showed red blood cell count (RBC) 4.46 × 10^12^/L, hemoglobin 111 g/L, ALT 53 U/L, AST 42 U/L, lactate dehydrogenase (LDH) 252 U/L. and serum potassium of 2.99 mmol/L. Twenty four hour urine electrolytes showed sodium 120.2 mmol/24 h and potassium 58.95 mmol/24 h. Cortisol levels at 8 a.m., 4 p.m., and 12 a.m. were 227, 114, and 59.10 nmol/L, respectively. ACTH levels at 8 a.m. and 4 PM were 10.37 and 8.81 ng/L, respectively. 1 mg DST: cortisol was 20.40 nmol/L, and ACTH was < 1.00 ng/L at 8 AM on the test day. Renal function, stool analysis, glycated hemoglobin, coagulation profile, thyroid function, plasma catecholamines and metabolites, and sex hormone levels were all within normal limits. Detailed results of the 24h urinary free cortisol, renin–angiotensin–aldosterone system (RAAS) testing in both supine and standing positions, saline infusion test, and captopril suppression test are summarized in Table 2. Electrocardiogram revealed sinus rhythm with T-wave abnormalities, QT interval prolongation, and prominent U waves. Positron emission tomography/computed tomography (PET/CT) with CXCR4-targeted imaging demonstrated multiple nodules in the left adrenal gland, suggestive of aldosterone-producing adenomas. The patient underwent laparoscopic left adrenalectomy, and postoperative histopathology confirmed an adrenal cortical adenoma. She was discharged without medication. During the two-month follow-up, she remained normotensive without the need for antihypertensive therapy. No recurrence of symptoms such as muscle pain or weakness was observed. Follow-up tests revealed normal liver function, cardiac enzyme levels, and serum potassium, indicating a favorable prognosis.

**Table 2:** Summary of endocrine function tests.

Laboratory test	Preoperative standard posture test of the renin-angiotensin-aldosterone system	
Supine		Upright
PRA (μIU/Ml)	< 0.50	0.65
PAC (ng/dL)	94.60	83.70
PAC: PRA	-	128.77
Laboratory test	Saline infusion test	
Before saline infusion-upright		After saline infusion-upright
PRA (μIU/ml)	1.31	0.95
PAC (ng/dL)	90.90	87.50
PAC: PRA	69.39	92.11
K (mmol/l)	3.64	3.66
Serum cortisol (nmol/l)	168.00	141.00
Laboratory test	Captopril challenge test	
Before captopril-upright		After captopril-upright
PRA (μIU/ml)	1.72	1.97
PAC (ng/dL)	81.60	93.40
PAC: PRA	47.44	47.41
K (mmol/l)	3.46	3.29
Laboratory test	24h urinary free cortisol	
First test		Second test
24h UV (ml)	1,500.00	1,800.00
Urinary cortisol (nmol/l)	61.15	37.30
24h UFC (μg/24 h)	88.70	64.90

PRA, plasma renin activity; PAC, plasma aldosterone concentration; K, serum potassium; 24h UV, 24-hour urine volume; 24h UFC, 24-hour urinary free cortisol.

The patient presented with recurrent pain and muscle weakness, which limited her daily activities significantly. She had visited multiple hospitals before, but no clear cause had been identified, leading to obvious anxiety. After admission to our hospital, the underlying cause was confirmed, and she received systematic treatment followed by further surgical intervention. At present, she has returned to normal daily life and expressed great relief and satisfaction with the treatment outcome.

## Discussion

The patient had recurrent muscle pain and generalized weakness, with markedly elevated CK, varying degrees of increased liver enzymes, and elevated Mb—findings consistent with RM. RM is a clinical syndrome caused by skeletal muscle cell breakdown (from multiple causes) that releases intracellular contents into the bloodstream, typically presenting with muscle pain, weakness, and dark urine. Laboratory findings typically show elevated CK, AST, ALT, LDH, and electrolyte disturbances. CK levels are often markedly increased, sometimes exceeding 10 times the upper normal limit (5). RM has diverse etiologies, broadly categorized into physical and non-physical muscle injuries. Severe cases progress rapidly, causing life-threatening complications or even death; thus, early diagnosis and timely intervention are crucial for disease control and improved outcomes.

In this case, the patient had episodes only after mild activity, with a striking feature of persistent severe hypokalemia during episodes. Notably, there were no typical predisposing factors like fever, trauma, infection, or drug toxicity. While RM is commonly associated with hyperkalemia (6), hypokalemia-induced RM is relatively uncommon (7). Her serum potassium remained markedly low with poor response to supplementation, suggesting a possible link to underlying potassium homeostasis disturbance, warranting further investigation into the etiology. Abnormal potassium distribution typically occurs in conditions like insulin administration or excess catecholamine secretion, but the patient had no insulin use history and normal catecholamine levels. For increased renal potassium loss, several renal conditions can impair tubular potassium reabsorption and cause excessive urinary potassium excretion, including the polyuric phase of AKI, early recovery after relief of urinary tract obstruction, renal tubular acidosis, and hereditary/acquired disorders such as Liddle, Gitelman, and Bartter syndromes. However, the patient had normal renal function; renal ultrasonography showed no urinary tract obstruction, and ABG indicated metabolic alkalosis. Genetic testing was negative with no pathogenic mutations. The patient had intermittently used hydrochlorothiazide-containing antihypertensives, which promote distal renal tubular potassium excretion and may cause hypokalemia. Notably, the patient had a prior history of hypertension, and 24h ambulatory BP monitoring demonstrated a reverse-dipper BP pattern, an abnormal circadian rhythm that suggests the possible presence of underlying endocrine hypertension. Subsequent adrenal contrast-enhanced computed tomography and endocrine evaluations supported the diagnosis of PA. In addition, drug-induced hypokalemia should also be considered. The patient had intermittently used antihypertensive medications containing hydrochlorothiazide for BP control, and thiazide diuretics can increase distal tubular sodium delivery and promote potassium excretion, thereby reducing serum potassium levels. Therefore, in this case, the potential role of hydrochlorothiazide in aggravating potassium loss and subsequently triggering or exacerbating RM cannot be ignored. However, because the patient used the medication irregularly, the exact timing and dosage of hydrochlorothiazide could not be reliably determined, making it difficult to establish a clear temporal correlation with episodes of hypokalemia and RM. Notably, after modification of the antihypertensive regimen, the patient was treated with spironolactone in combination with diltiazem hydrochloride for BP control; during the first follow-up, serum potassium levels remained within the normal range and no recurrence of RM was observed, further suggesting that thiazide diuretics may have played an important triggering or exacerbating role in the development of hypokalemia and RM. Furthermore, no common precipitating factors for RM were identified in this patient, including strenuous exercise, trauma, infection, alcohol abuse, or the use of myotoxic medications. Although the two episodes occurred after activities such as hiking and moving routine household items, the patient reported that the level of exertion was mild and comparable to routine physical activity, which is generally insufficient to independently induce RM; therefore, severe hypokalemia likely played a more critical role in its development.

PA is an endocrine disorder characterized by excessive aldosterone production due to adrenal cortical dysfunction, resulting in suppression of the renin-angiotensin system. While hypertension is typically the initial and most prominent clinical feature (8), hypokalemia is also a key manifestation. However, studies have shown that only approximately 32.12% of patients with PA exhibit hypokalemia (9). In rare instances—such as the present case—hypokalemia-induced RM may be the first manifestation, highlighting the importance of considering PA in the differential diagnosis of unexplained hypokalemia-related muscle injury. The underlying mechanisms of hypokalemia-induced RM are multifactorial and may include: reduced cellular membrane stability due to potassium deficiency, leading to increased susceptibility of muscle cells to damage; hypokalemia-induced vasoconstriction, which impairs skeletal muscle perfusion; and impaired glucose metabolism within muscle cells resulting from potassium depletion, leading to reduced intracellular energy supply and subsequent muscle fiber necrosis (10). These pathological processes may be further aggravated by concurrent metabolic disturbances, such as metabolic alkalosis.

After discharge from the first hospital where she was treated, the patient did not receive potassium supplementation or undergo regular follow-up. The recurrence of RM following routine physical activity—such as relocating daily items—suggests that hypokalemia persisted during this period. This highlights hypokalemia as a significant risk factor for RM. Previous studies have demonstrated that severe hypokalemia (serum potassium < 2.5 mmol/L) markedly increases the risk of developing RM (11). Consistent with this, in the present case, even mild exertion was sufficient to induce RM. The severity of RM has been shown to correlate with both the degree of hypokalemia and the presence of underlying medical conditions (7). In patients with hypokalemia-induced RM, the rupture of muscle cells leads to the release of intracellular potassium into the bloodstream, which may result in a transient elevation in serum potassium levels. This can obscure the underlying systemic potassium deficiency (12). If hypokalemia is not promptly recognized and corrected, progressive potassium depletion—even in the context of routine daily activities—can aggravate muscle breakdown and result in serious complications. Furthermore, in cases of PA-associated hypokalemia, elevated aldosterone levels promote excessive renal potassium excretion, rendering conventional potassium supplementation less effective. Therefore, early and aggressive potassium replacement is essential for effective management. Following potassium supplementation, fluid resuscitation, and discontinuation of potassium-depleting diuretics, the patient's serum potassium levels stabilized, and RM resolved. Upon discharge, spironolactone was prescribed to counteract aldosterone excess, and Diltiazem Hydrochloride was administered for BP control. Subsequent follow-up revealed stable serum potassium levels with no recurrence of RM. Previous studies have also reported similar cases in which patients developed severe limb weakness after the addition of thiazide diuretics, and subsequent evaluation confirmed PA with severe hypokalemia leading to RM (13). This observation highlights the important clinical implications of antihypertensive drug selection in patients with confirmed or suspected PA. Although thiazide diuretics are widely recommended as first-line antihypertensive agents, they may significantly aggravate potassium loss in patients with mineralocorticoid excess and should therefore be used with caution, particularly when hypokalemia has not yet been corrected or PA has not been excluded. In such patients, calcium channel blockers, angiotensin-converting enzyme inhibitors, angiotensin II receptor blockers, or mineralocorticoid receptor antagonists such as spironolactone or eplerenone may represent safer antihypertensive options.

In this case, multiple ABG revealed metabolic alkalosis. Once the diagnosis of PA was confirmed, the metabolic alkalosis was attributed to enhanced sodium-hydrogen exchange in the distal renal tubules secondary to hypokalemia (14). This case also highlights the importance of selecting the appropriate potassium when correcting hypokalemia in patients with PA-induced RM. Given the frequent presence of metabolic alkalosis in PA, potassium chloride is the preferred supplement. Potassium citrate should be avoided, as it may exacerbate the alkalosis. Early and aggressive fluid resuscitation is the cornerstone of RM management to prevent AKI. Adequate volume expansion promotes renal tubular perfusion, dilutes nephrotoxic substances such as Mb, and ensures sufficient renal blood flow. When CK levels >5,000 U/L, the risk of AKI significantly increases. Standard treatment includes prompt and sufficient fluid replacement and urine alkalinization, aiming to maintain urine output at 1–3 mL/kg/h (maximum of 300 ml/h). This approach helps dilute urinary toxins, prevents the accumulation of Mb and other nephrotoxic substances in the renal parenchyma, and maintains a urine pH >6.5 to reduce the risk of Mb precipitation in renal tubules. When CK levels are < 5,000 U/L, the risk of AKI is considered low, and aggressive fluid resuscitation or urine alkalinization is generally not necessary (6). This is particularly important in patients who already exhibit metabolic alkalosis, in whom alkalinization therapy should be avoided to prevent further acid-base disturbances. Continuous monitoring of CK levels and urine output is essential. If oliguria or anuria persists despite adequate fluid resuscitation, renal replacement therapy (RRT) should be considered. In this case, the patient was ultimately diagnosed with PA caused by an aldosterone-producing adenoma. Following laparoscopic left adrenalectomy, the patient was discharged without the need for further medications. During follow-up, both BP and serum potassium levels remained within normal ranges, and no recurrent symptoms such as limb weakness or muscle pain were reported. The absence of recurrent RM further confirmed PA as the underlying cause of RM in this patient.

## Conclusions

This case highlights that RM may serve as an early clinical indicator of underlying systemic conditions. Early recognition and comprehensive etiological evaluation are essential for timely intervention and improved patient outcomes. Although PA-induced secondary RM is uncommon, it is frequently underdiagnosed or misdiagnosed. Clinicians should consider evaluating the RAAS function and performing adrenal imaging in patients with the following features: hypertension; recurrent RM, particularly in the presence of spontaneous hypokalemia; inadequate response to potassium supplementation; or unexplained metabolic alkalosis on ABG. While adrenal-related RM is rare, it has a clearly defined and reversible etiology. Once diagnosed, appropriate surgical intervention can result in excellent clinical outcomes. In patients with hypertension—particularly those with recurrent limb weakness or a history of hypokalemia—serum electrolyte levels should be routinely assessed before initiating or adjusting antihypertensive therapy; if hypokalemia is detected, further evaluation should be undertaken to determine the underlying cause and avoid overlooking potential endocrine hypertension or drug-related adverse effects.

## Data Availability

The raw data supporting the conclusions of this article will be made available by the corresponding author upon reasonable request, subject to patient privacy and ethical considerations.
